# Long-range ballistic transport of Brown-Zak fermions in graphene superlattices

**DOI:** 10.1038/s41467-020-19604-0

**Published:** 2020-11-13

**Authors:** Julien Barrier, Piranavan Kumaravadivel, Roshan Krishna Kumar, L. A. Ponomarenko, Na Xin, Matthew Holwill, Ciaran Mullan, Minsoo Kim, R. V. Gorbachev, M. D. Thompson, J. R. Prance, T. Taniguchi, K. Watanabe, I. V. Grigorieva, K. S. Novoselov, A. Mishchenko, V. I. Fal’ko, A. K. Geim, A. I. Berdyugin

**Affiliations:** 1 Department of Physics and Astronomy, University of Manchester, Manchester, M13 9PL UK; 2 National Graphene Institute, University of Manchester, Manchester, M13 9PL UK; 3 Department of Physics, University of Lancaster, Lancaster, LA1 4YW UK; 4 National Institute for Materials Science, Ibaraki, 305-0044 Japan

**Keywords:** Materials science, Electronic properties and materials

## Abstract

In quantizing magnetic fields, graphene superlattices exhibit a complex fractal spectrum often referred to as the Hofstadter butterfly. It can be viewed as a collection of Landau levels that arise from quantization of Brown-Zak minibands recurring at rational (*p*/*q*) fractions of the magnetic flux quantum per superlattice unit cell. Here we show that, in graphene-on-boron-nitride superlattices, Brown-Zak fermions can exhibit mobilities above 10^6^ cm^2^ V^−1^ s^−1^ and the mean free path exceeding several micrometers. The exceptional quality of our devices allows us to show that Brown-Zak minibands are 4*q* times degenerate and all the degeneracies (spin, valley and mini-valley) can be lifted by exchange interactions below 1 K. We also found negative bend resistance at 1/*q* fractions for electrical probes placed as far as several micrometers apart. The latter observation highlights the fact that Brown-Zak fermions are Bloch quasiparticles propagating in high fields along straight trajectories, just like electrons in zero field.

## Abstract (web summary)

Here, the authors show that Brown-Zak fermions in graphene-on-boron-nitride superlattices exhibit mobilities above 10^6^ cm^2^/V s and micrometer scale ballistic transport.

## Introduction

Van der Waals assembly offers a possibility to create materials by stacking atomically-thin layers of different crystals^1–3^. One of the simplest and most studied van der Waals heterostructures is graphene encapsulated between two hexagonal boron nitride (hBN) crystals. The encapsulation protects graphene from extrinsic disorder^4,5^, allowing ultra-high electronic quality and micrometer-scale ballistic transport often limited only by edge scattering^6,7^. A special case of encapsulated graphene heterostructures is graphene superlattices, where crystallographic axes of graphene and hBN are intentionally aligned. A small (1.8%) mismatch between graphene and hBN crystal lattices results in a periodic moiré potential acting on charge carriers in graphene and leading to the formation of electronic minibands^1–3,8–17^.

A relatively large (∼14 nm) periodicity of graphene-on-hBN superlattices has also made it possible to study the regime of Hofstadter butterflies, which requires the magnetic flux *ϕ* per superlattice unit cell to be comparable to the flux quantum *ϕ*_0_ in experimentally-accessible magnetic fields *B*. At *B* = *B*_p/q_ corresponding to *ϕ* = *ϕ*_0_ *p*/*q*, where *p* and *q* are integer, the translational symmetry of the electronic system is restored (despite the presence of a quantizing magnetic field) and the superlattice’s electronic spectrum can again be described in terms of Bloch states^18–29^, just like in *B* = 0. These high-field Bloch states are characterized by their own miniband spectra^25–29^ different from the zero-*B* spectrum of graphene-on-hBN superlattices. The associated quasiparticles are referred to as Brown-Zak (BZ) fermions. According to the group-theory analysis, an electronic spectrum for each realization of BZ fermions should have an additional *q*-fold degeneracy^18–27,30,31^ (that is, contains *q* equivalent mini-valleys). This degeneracy is additional to the 4-fold spin and valley degeneracy of graphene’s original spectrum. Importantly, BZ fermions are Bloch quasiparticles, like electrons in solids or Dirac fermions, and, at *B* = *B*_p/q_, they move through the superlattice as if the applied field is zero. Away from these exact values, BZ fermions experience an effective magnetic field *B*_eff_ = *B* − *B*_p/q_ (refs. ^18–27,30,31^).

## Results

### Experimental devices and measurement setup

**Fig. 1:**
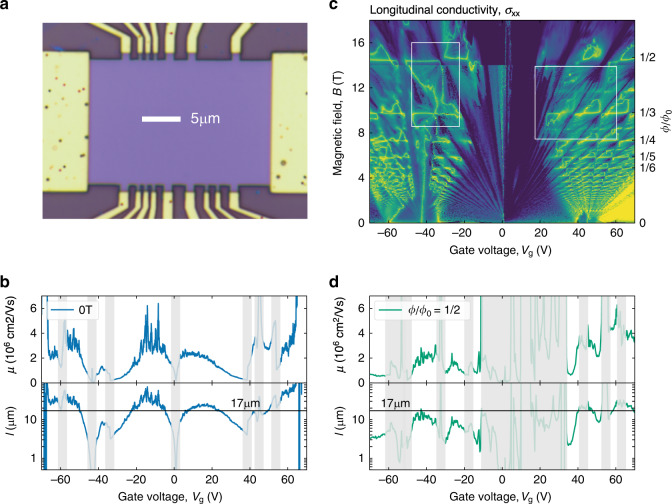
High-quality graphene superlattices and their transport properties. **a** Optical micrograph of one of our devices (D1; twist angle *θ* between graphene and hBN of about 0.4°). The Hall bar is seen in violet with golden electrical contacts. **b** Mobility and mean free path for D1 measured at zero *B* and 10 mK. Semitransparent vertical strips indicate the doping regions around NPs and vHS where *n* could not be extracted directly from Hall measurements and charge inhomogeneity also plays a role. To calculate *μ* and *l* within the shaded regions, we assumed a constant gate capacitance and linearly extrapolated the *n*(*V*_g_) dependences found sufficiently far from NPs and vHS (Supplementary Note 3). The noisy behavior at large values of *μ* and *l* arises from *ρ*_xx_ becoming small (∼1 Ohm, about 4 orders of magnitude smaller than that at the NPs). The horizontal black line indicates the device width *W*. **c** σ_xx_(*V*_g_, *B*) measured by sweeping *V*_g_ and varying *B* in small steps of 40 mT. *T* = 10 and 250 mK below and above 14 T, respectively. Indigo-to-yellow colors: Log scale truncated between 38 nS and 16 mS for *B* < 14 T and between 4 nS and 0.4 mS above 14 T. White rectangles: these regions are shown in finer detail in Figs. 3 and 4. **d** Same as in (**b**) but for *ϕ*/*ϕ*_0_ = 1/2 (*B* ≈ 15 T); *T* = 250 mK. In addition to NPs and vHS, the gray strips also cover a wide region of the quantum Hall regime (|*V*_g_ | < 20 V), which is dominated by large cyclotron gaps in the main graphene spectrum. The transport data used to calculate *μ* and *l* in (**b**) and (**d**) are shown in Supplementary Note 3.

We report the electronic properties of BZ fermions with different *p* and *q* using high-quality graphene superlattices. These devices were fabricated using the standard dry transfer procedures, where the studied graphene crystal was carefully aligned with one of the encapsulating hBN crystals using the crystallographic edges^11^. The alignment was verified by Raman spectroscopy^32^ prior to encapsulation with the second hBN crystal. The latter was intentionally misaligned to avoid competing moiré patterns^33–35^. The assembled stacks were placed on an oxidized Si wafer, which allowed us to apply the back-gate voltage *V*_g_ to control the carrier density *n*. We studied six devices that were shaped into the multiterminal Hall bar geometry and had the main channel widths *W* ranging from 2 to 17 μm (see Fig. 1a and Supplementary Note 1). The devices were first characterized by measuring their longitudinal resistivity *ρ*_xx_ in zero *B* and Hall resistivity *ρ*_xy_ in small non-quantising *B* below 0.1 T. The latter enabled us to find the *n*(*V*_g_) dependences, except for gate voltages close to the neutrality points (NPs) and van Hove singularities (vHS), where *ρ*_xy_ reversed its sign and could no longer be described by the standard dependence *ρ*_xy_ = *B*/*ne* (*e* is the electron charge). All our devices exhibited very high carrier mobilities *μ* = (*ρ*_xx_*ne*)^−1^ of the order of 10^6^ cm^2^ V^−1^ s^−1^, which were still somewhat reduced by edge scattering because of finite *W* (Fig. 1b). We corroborated the high quality of our devices using transverse magnetic focusing measurements (Supplementary Note 2). In the main text, we focus on two large-width devices (D1 and D2) exhibiting highest *μ*.

Figure 1c shows a map of the longitudinal conductivity $\sigma _{{\mathrm{xx}}} = \rho _{{\mathrm{xx}}}/(\rho _{{\mathrm{xx}}}^2 + \rho _{{\mathrm{xy}}}^2)$ as a function of *V*_g_ and *B* in fields up to 18 T. The main features on such maps have been well understood in terms of the Hofstadter spectrum for Dirac fermions in moiré superlattices^11–17,22^. One can see numerous Landau levels (LLs) fanning out from the main and secondary NPs (miniband edges) which are located at *V*_g_ near 0 and ±45 V, respectively. There are also pronounced funneling features near van Hove singularities at *V*_g_ ≈ −60, −35, +40 and +55 V in both hole (−) and electron (+) parts of the spectrum. Another important attribute of the map is horizontal yellow streaks that occur at *ϕ*/*ϕ*_0_ *p*/*q* (Fig. 1c). If temperature *T* is increased above 100 K so that Landau quantization is strongly suppressed, the horizontal streaks become the only dominant feature on such transport maps^28,29^. In the high-*T* regime, the streaks represent oscillations in both *ρ*_xx_ (*B*) and *ρ*_xx_ (*B*) at a constant *V*_g_. Their 1/*B* frequency is independent of *n*, and they were named BZ oscillations^28^. The horizontal streaks are seen in Fig. 1c correspond to maxima in σ_xx_ and zeros in *ρ*_xx_ and, as explained in the introduction, reflect the recovery of translational symmetry (*p* flux quanta penetrate through *q* superlattice unit cells) and the emergence of Bloch states experiencing zero *B*_eff_. Along each horizontal streak, one can find numerous NPs and vHS, which reflect different realizations of BZ fermions at each *B*_p/q_. Landau mini-fans radiate from these NPs in both directions along the *B* axis (Fig. 1c), representing Landau quantization of BZ electronic spectra by non-zero *B*_eff_ (see below)^18–29^. Another notable feature of the shown σ_xx_ map is a repetitive triangular-like pattern seen most clearly between 2 and 12 T, especially for positive *V*_g_. The yellow triangles are made of horizontal streaks at zero *B*_eff_, vertical streaks emerging from NPs for BZ fermions and slanted streaks originating from vHS.

### Ballistic transport

The transport properties of BZ fermions can be analyzed in the same way as in Fig. 1b for Dirac fermions. The results are plotted in Fig. 1d for the case of *ϕ*/*ϕ*_0_ = 1/2 and show that, away from NPs and vHS, BZ fermions in our devices exhibit *μ* reaching a few 10^6^ cm^2^ V^−1^ s^−1^. This is comparable to *μ* of Dirac fermions in zero *B*. Another important characteristic of charge carrier transport is the mean free path *l*. It can be evaluated for both Dirac and BZ fermions, using the standard expression $\sigma _{{\mathrm{xx}}} = g\frac{{e^2}}{h}\left( {\frac{{k_{\mathrm{F}}l}}{2}} \right)$ where *h* is the Planck constant, *k*_F_ is the Fermi momentum and *g* is the degeneracy. In zero *B*, *g* = 4 because of the spin and valley degeneracy of Dirac fermions. BZ fermions are expected to have an additional, mini-valley degeneracy^18–27,30,31^, which is equal to *q* (that is, *g* = 8 for the case of Fig. 1d). Using these *g*, we calculated *l* as shown in the bottom panels of Fig. 1b, d. The mean free path in zero *B* reached > 20 μm, implying that ballistic transport in our devices is limited by *W* rather than impurity scattering. For some realizations of BZ fermions, their mean free path also exceeds 10 μm, marginally smaller than *l* for Dirac fermions (cf. Fig. 1b, d). Supplementary Note 4 provides similar analysis for *μ* and *l* at other values of *q*.

**Fig. 2:**
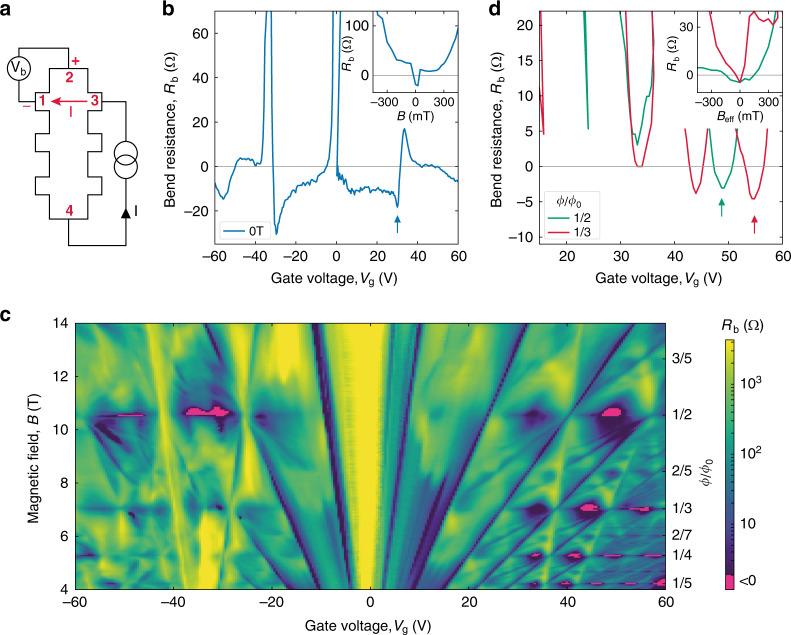
Ballistic transport of BZ fermions over micrometer distances. **a** Schematic of bend resistance measurements. Current *I* is applied between contacts 3 and 4, and voltage *V*_b_ is measured between 2 and 1, yielding the bend resistance, *R*_b_ = *V*_b_/*I*. The voltage is positive for diffusive transport but becomes negative, if charge carriers move directly from current injecting contact 3 into voltage probe 1 (as shown by the red arrow). **b** Bend resistance for Dirac fermions in zero *B* (device D2 with *W* = 4 μm and *θ* ≈ 0°). Inset: *R*_b_(*B*) taken at the minimum indicated by the arrow in the main plot. **c** Map *R*_b_(*V*_g_,*B*) for the same device. *B* was changed in steps of 50 mT. Pockets of negative *R*_b_ appear along *ϕ*/*ϕ*_0_ = 1/*q* and are seen in magenta. **d** Cross-sections from (**c**) for *q* = 2 and 3. The inset shows sign reversals in *R*_b_ plotted as a function of *B*_eff_ = *B* – *B*_p/q_ for the minima marked by the color-coded arrows. *T* = 2 K for all the plots.

We corroborate the existence of ballistic BZ fermions using so-called bend resistance geometry^5,36^ sketched in Fig. 2a. The geometry allows one to detect if charge carriers can move ballistically over the entire channel width *W*, along straight trajectories connecting current and voltage contacts (see caption of Fig. 2a). If this is the case, ballistic transport gives rise to the negative sign of the bend resistance *R*_b_ (ref. ^36^) in contrast to its conventional, positive sign for diffusive (ohmic) transport. As expected from very long *l* of Dirac fermions, *R*_b_ was negative in zero *B* everywhere away from NPs and vHS (Fig. 2b), confirming further the high quality of our superlattice devices. Finite *B* bend Dirac fermion trajectories and, as expected, *R*_b_ rapidly reversed its sign^36^ with increasing *B* (inset of Fig. 2b). Remarkably, our devices exhibited negative *R*_b_ also in high *B* = *B*_p/q_, thus revealing straight trajectories over distances of several μm (Fig. 2c, d and Supplementary Note 5). For comparison, the corresponding map of *ρ*_xx_ is provided in Supplementary Note 6, which shows that the measured longitudinal resistance always remained positive. The profound negative pockets in *R*_b_(*V*_g_,*B*) appeared only around *B* corresponding to *ϕ*/*ϕ*_0_ = 1/2, 1/3, 1/4 and 1/5. This is the regime where the existence of BZ fermions, experiencing zero *B*_eff_, was previously inferred^28,29^ from maxima in σ_xx_ and zeros in *ρ*_xy_. The possibility of ballistic transport of BZ fermions was also suggested using numerical simulations^15^. The observed negative *R*_*b*_ prove the previous conjectures unequivocally. The negative pockets in Fig. 2c are located between NPs and vHS for BZ fermions, similar to the case of Dirac fermions. Away from *B* = *B*_p/q_, non-zero *B*_eff_ bends the BZ fermion trajectories and the negative signal disappeared (inset of Fig. 2d), again like in the case of other Bloch quasiparticles (electrons and Dirac fermions). Our devices exhibited long-range ballistic transport of BZ fermions only for unit fractions, *ϕ*_0_/*q*. For example, Fig. 2c reveals a pronounced set of negative *R*_b_ pockets at *ϕ*/*ϕ*_0_ = 1/5 but no negative signal was observed for 2/5 and 3/5. This behavior probably stems from lower *μ* of BZ fermions with *p* > 1, which can be attributed to their larger effective masses^29^.

### Lifting degeneracy in BZ minibands

**Fig. 3:**
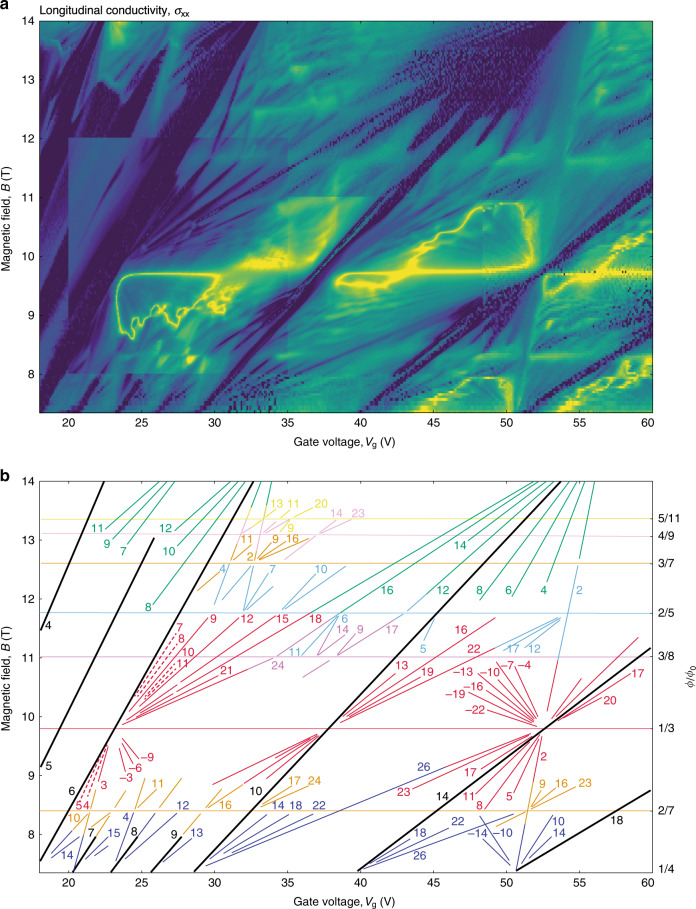
Landau quantization in BZ minibands at 10 mK. **a** High-resolution map σ_xx_(*V*_g_, *B*) for the electron-doped region indicated in Fig. 1c by the white rectangle (device D1). To better resolve LLs around *q* = 3, *B* was changed in steps of 10 to 20 mT whereas data in other regions were acquired using 40 to 80 mT steps. This has resulted in the contrast discontinuities seen in the map. Log color scale: indigo (230 nS) to yellow (7.8 mS) for, the entire map. **b** Minima from (**a**) are shown schematically. The color-coded numbers are the filling factors for the corresponding LLs. Thick black lines correspond to the main sequence of LLs for graphene’s Dirac spectrum (spin and valley degeneracy lifted). The green, red, navy, blue, orange, magenta, pink, and yellow lines correspond to *q* = 2, 3, 4, 5, 7, 8, 9, and 11, respectively. Dashed red lines: minima due to lifted mini-valley degeneracy.

**Fig. 4:**
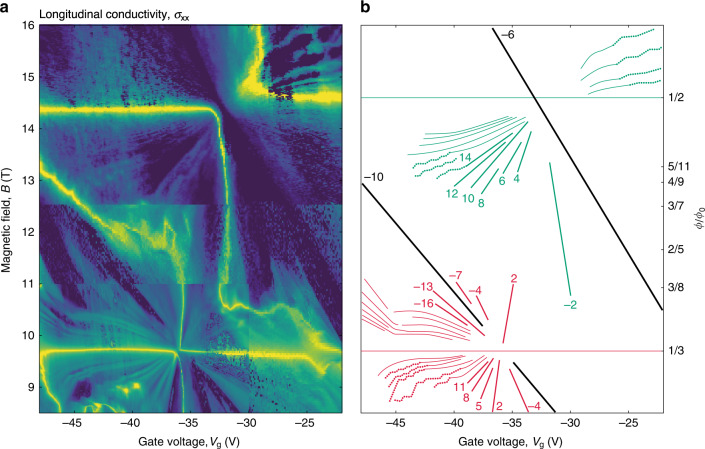
Anomalous behavior of Landau levels in BZ minibands. **a** High-resolution σ_xx_ (*V*_g_, *B*) for the hole-doped region marked in Fig. 1c (device D1). The measurements below *B* = 12.5 T were carried out at 10 mK, and at 250 mK in the fields above. *B* was applied in steps of 16 and 40 mT above and below 12.5 T, respectively, except for the low left corner where the data were acquired at higher resolution of 10 mT (seen as discontinuities in the contrast). Log scale: indigo (310 nS) to yellow (0.3 mS). **b** Schematics for the conductance minima found in (**a**). The same color coding as in Fig. 3b. The solid lines indicate LLs evolving as expected, linearly in *B* and *V*_g_. The thin curves indicate the anomalous bending whereas the dotted curves show the staircase-like evolution observed for some LLs. All the anomalous features were highly reproducible and, for example, did not depend on the step size in *B*.

The high mobility of BZ fermions resulted in their Landau quantization in small *B*_eff_ < 1 T, which allowed us to find experimentally the spectral degeneracy *g* for different *p/q*. To this end, Fig. 3a shows a high-resolution map of σ_xx_ between *ϕ*/*ϕ*_0_ = 1/2 and 1/4 (part of Fig. 1c). One can clearly see many Landau mini-fans originating from NPs for different realizations of BZ fermions. For example, there are three profound mini-fans spreading from *B* ≈ 9.7 T (*ϕ*/*ϕ*_0_ = 1/3) for both negative and positive *B*_eff_. For clarity, the conductivity map in Fig. 3a is replotted in Fig. 3b by tracing all well-defined minima in σ_xx_. Each minimum can be described by its integer filling factor *ν*, which we calculated from the minimum’s slope. This representation of the Hofstadter spectrum, where LLs are plotted as a function of *n* or *V*_g_ rather than energy, is usually referred to as the Wannier diagram^11–17,21,22^. In the diagram of Fig. 3b, one can identify LLs for BZ-fermion realizations at *q* = 2, 3, 4, 5, 7, 8, 9, and 11, and for *p* = 1, 2, 3, 4, and 5. The difference in *ν* between the nearest LLs yields directly their *g*. For example, all LLs found for *ϕ*/*ϕ*_0_ = 1/2 were separated by Δ*ν* = 2 whereas those at *ϕ*/*ϕ*_0_ = 1/3 and 1/4 by Δ*ν* = 3 and 4, respectively (see Fig. 3b). Therefore, the observed degeneracies were equal to *q*. Note that the measured Hall conductance also exhibited quantized values in steps of *qe*^2^/*h* (Supplementary Note 7). Examining the Wannier diagram further, we find that Δ*ν* was equal to *q*, independently of numerator *p* and for all LLs stemming from the same *B*_p/q_. This observation agrees with the theoretical expectation that there should be *q* equivalent mini-valleys for each realization of BZ fermions^18–27,30,31^. The agreement takes into account that both spin and valley degeneracies for BZ fermions were lifted by exchange interactions^14^, as in the case of the main Dirac spectrum with its clearly lifted degeneracies (see the LLs marked in black in Fig. 3b). Therefore, the total degeneracy for BZ minibands in Fig. 3 was *g* = 4*q*. This is further supported by our measurements at a relatively high *T* of 2 K that suppressed LLs with lifted spin and valley degeneracies (Supplementary Note 6). The same behavior (Landau mini-fans exhibiting the sequence Δ*ν* = *q*) was also observed in other parts of the Wannier diagram for both electron and hole doping (see, e.g., Fig. 4).

There is, however, one notable exception, which was observed for *ϕ*/*ϕ*_0_ = 1/3 (left part of the Wannier diagram in Fig. 3b; dashed lines). In this case, LLs of BZ fermions with *q* = 3 are separated only by Δ*ν* = 1 so that all consecutive LLs from 3 to 12 are present on the fan diagram. The indicated minima in σ_xx_ were rather fragile, being rapidly smeared by *T* or excitation current (Supplementary Note 7). Landau levels with Δ*ν* = 1 at *ϕ*/*ϕ*_0_ = 1/*q* do not exist within the single-particle Hofstadter-Wannier model^16,17,21,22^. Such ‘extra’ LLs have been observed previously and referred to as the fractional Bloch quantum Hall effect (FBQHE)^16^ or symmetry-broken Chern insulator (SBCI)^17^. Theoretically, the many-body states can arise from interaction effects that lift the mini-valley degeneracy of BZ fermions. This may happen via mini-valley mixing due to the formation of charge-density waves (commensurate with the magnetic superlattice composed of *q* unit cells of the underlying moiré pattern) or a Wigner crystal^37^ (states localized in a part of the magnetic supercell). Alternatively, the fully lifted degeneracy may occur through spontaneous mini-valley polarization of BZ LLs, analogous to spin/valley ferromagnetism in graphene^14^. In the latter case, the BZ fermion states localize in the momentum space around one of the mini-valleys but remain delocalized across the magnetic supercell.

## Discussion

Some Landau mini-fans exhibited highly anomalous behavior at low *T*, which cannot be understood either within the Hofstadter-Wannier model or by considering LLs of non-interacting BZ fermions and, to the best of our knowledge, was never reported before. From either perspective, individual LLs must evolve linearly in *V*_g_ and *B*, as indeed seen in most cases (e.g., Fig. 3). This is because the density of states on each BZ-fermion LL is proportional to *B*_eff_ = *B* −*B*_p/q_. Also, the long (∼300 nm) distance from graphene to the gate in our devices makes quantum capacitance corrections^38,39^ negligible, leading to the *n* ∝ *V*_g_ dependence. Unexpectedly, we found that some Landau fans of BZ fermions exhibited bending and staircase-like features. Figure 4 shows examples of such behavior for the case of hole doping. Both bending and staircases appear away from NPs, in the regions close to BZ fermions’ vHS. The observed bending towards the gate-voltage axis indicates that, in addition to the visible LLs, there can be some other electronic states that are populated in parallel. Because electron-electron interactions clearly play a significant role under the reported experimental conditions (e.g., they lift all the spectral degeneracies), it is reasonable to expect that interactions are also involved in the described anomalies. However, the usual suspects, such as negative compressibility cannot possibly explain our findings (in the latter case, LLs would bend toward the *B* axis). The anomalous behavior is possibly due to an interplay of BZ-fermion LLs with quantized states originating from nearby vHS, which would lead to redistribution of charge carriers between states with the light and heavy effective masses. One possible scenario is localization of electrons within some parts of the magnetic supercell, because its size becomes notably larger than the magnetic length at *B* > 10 T (Wigner crystallization mentioned above). Unfortunately, we could not find any additional features that would enable us to decipher origins of the described anomalies and, therefore, have to leave them for further investigation.

## Methods

### Devices fabrication

The reported graphene-on-hBN superlattices were assembled using the standard dry transfer procedures and polydimethylsiloxane (PDMS) stamps coated with a polypropylcarbonate (PPC) layer^7^. The thicknesses of the used hBN crystals was between 20 and 70 nm. After the trilayer stack was assembled, we used the standard electron beam lithography and reactive ion etching to define trenches for electrical contacts. Cr/Au films were evaporated into the trenches. Finally, using ion etching again, the trilayer stack was shaped into a Hall bar mesa.

### Electrical measurements

For electrical measurements, the standard low-frequency lock-in technique was employed. At temperatures below 100 mK, we used excitation currents of ∼10 nA, and between 0.1 to 1 μA at higher *T*. The carrier concentration was controlled by applying a DC gate voltage between graphene and the silicon substrate.

## Supplementary information

Supplementary Information

## Data Availability

The data that support the findings of this study are available from the corresponding author upon reasonable request.
