# Supplementary material for: Long-range ballistic transport of Brown-Zak fermions in graphene superlattices
Source: Nat Commun. 2020 Nov 13;11:5756. doi: 10.1038/s41467-020-19604-0 (PMC7666116; doi:10.1038/s41467-020-19604-0)
Supplement: Supplementary file 1 — Supplementary Information [file 41467_2020_19604_MOESM1_ESM.pdf]

Supplementary information

Long-range ballistic transport of Brown-Zak fermions in graphene superlattices

J. Barrier *et. al.*

Supplementary Note 1

Studied devices. We studied six different superlattice devices as summarized in Supplementary Table 1. Here W refers to the device width, C_g is its gate capacitance and θ is the angle between the crystallographic axes of hBN and graphene. The latter was calculated from the measured period of BZ oscillations[1].

Supplementary Table 1

Device	W (μm)	θ (°)	C_g (μF/m^2)
D1	17	0.4	101
D2	4	0	98
D3	3	0.2	104
D4	3.2	0.5	96
D5	2	0.2	106
D6	2	0.3	98

Supplementary Note 2

Magnetic focusing in graphene superlattices. As a further confirmation of the devices' high quality, we report transverse magnetic focusing (TMF) experiments at low B (Supplementary Fig. 1). The observation of resistance oscillations due to TMF confirms that Dirac fermions travel ballistically across the device[2,3], forming skipping orbits extending over hundreds of superlattice unit cells. TMF measurements are also known to provide information about the Fermi surface topography in clean metals, including graphene superlattices[2,3]. Our TMF results are in agreement with those reported previously[2,4].

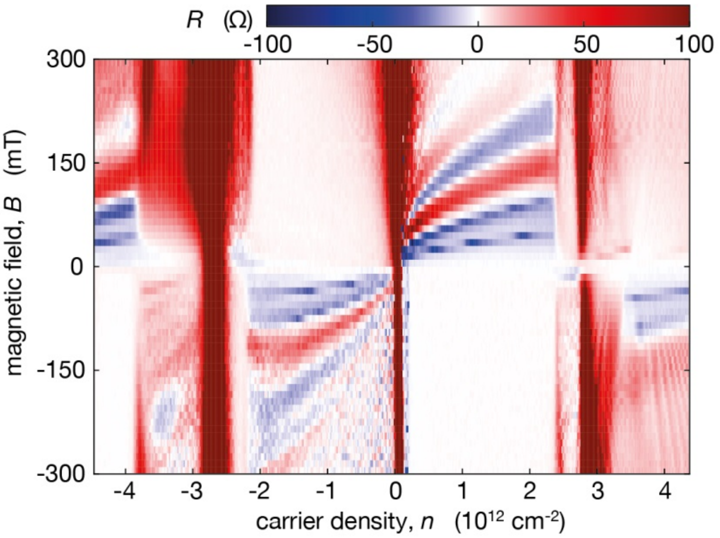

Supplementary Figure 1 | Transverse magnetic focusing. Measurements using contacts separated by 1.5 μm (closest contacts for device D1 pictured in Fig. 1a of the main text); T = 10 mK.

Supplementary Note 3

Determining mobility and mean free path of BZ fermions. To evaluate the mobility of Brown-Zak fermions, we use the standard formula $\mu = \sigma_{xx}/n_{BZF}e$ where n_{BZF} is the carrier density of BZ fermions and $\sigma_{xx} = 1/\rho_{xx}$. Note that the latter expression is exact at $\phi = \phi_0 p/q$ (that is, it does not contain ρ_{xy} because the effective magnetic field B_{eff} acting on BZ fermions is zero). To determine n_{BZF} for a given V_g, we first used Hall measurements at small fields $B \leq 0.1$ T to determine the geometrical capacitance. Then, using longitudinal conductivity maps around the p/q fractions, we identified positions of the neutrality points (NPs) as V_g into which Landau mini-fans converged (see Fig. 1c and Fig. 3a of the main text). Finally, vHS were identified from Hall effect measurements as

V_g where ρ_{xy} changed its sign without exhibiting mini-fans (Supplementary Fig. 2b). As n_{BZF} varies linearly across NPs and exhibits jumps at vHS, the known geometrical capacitance allowed us to reconstruct $n_{BZF}(V_g)$ as shown in Supplementary Fig. 2a.

The mean free path l was calculated using the standard formula $\sigma_{xx} = g\frac{e^2}{h}(\frac{k_F l}{2})$ where the Fermi wave vector $k_F = (4\pi n_{BZF}/g)^{1/2}$ also depends on the BZ-fermion degeneracy g. The final expression reads

$$l = \frac{2}{\rho_{xx}}\frac{\hbar}{e^2}\sqrt{\frac{\pi}{g\, n_{BZF}}} \quad (S1)$$

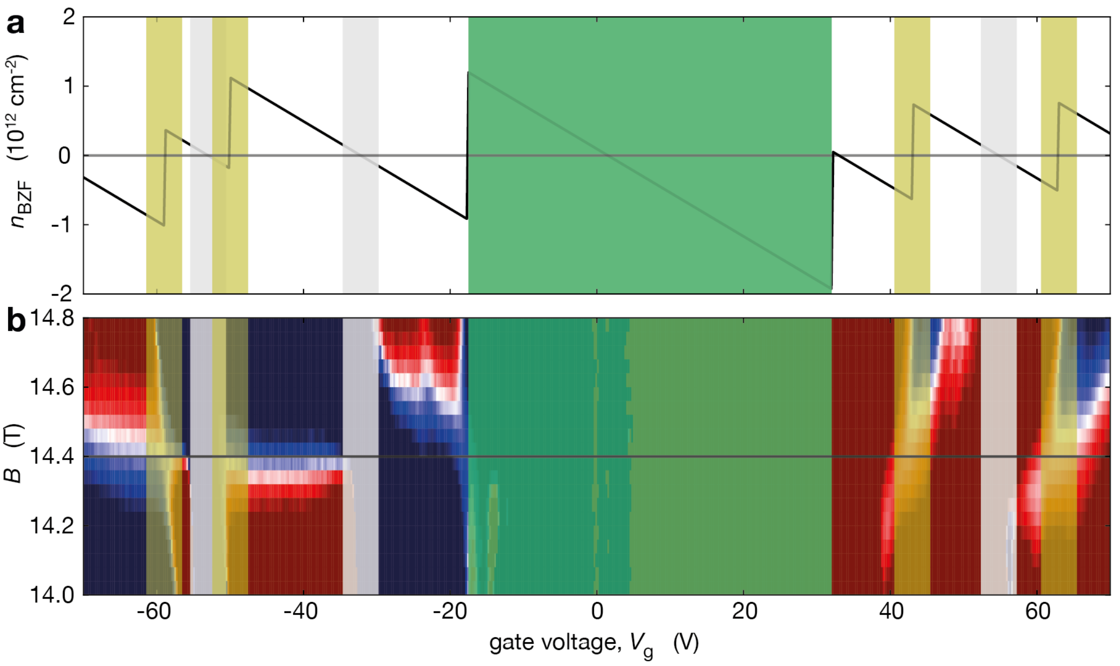

Supplementary Figure 2 | Evaluating density of BZ fermions. a, Dependence of n_{BZF} on gate voltage at $\phi/\phi_0 = 1/2$ for device D1. **b**, Measured maps for the Hall resistivity around $\phi/\phi_0 = 1/2$. Colour scheme: blue and red represent negative and positive ρ_{xy}, respectively. Regions around NPs are indicated by the grey semi-transparent strips. The yellow strips mark vHS. The central green area covers the region dominated by the quantum Hall effect of Dirac fermions from the main graphene spectrum (see Fig. 1c of the main text).

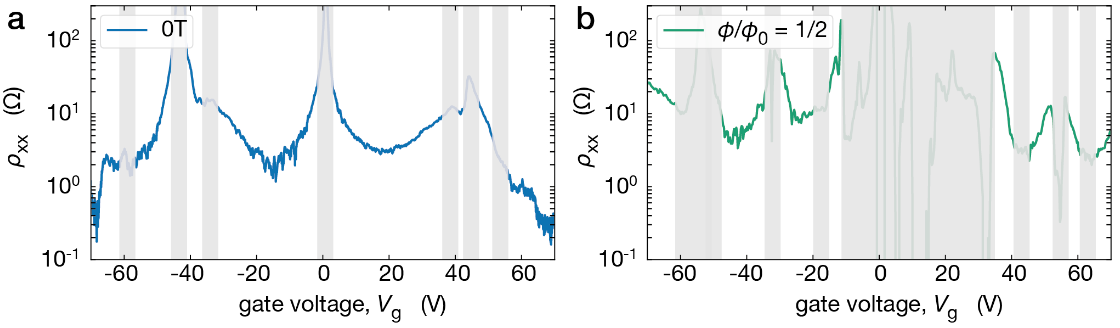

Supplementary Figure 3 | Longitudinal resistivity ρ_{xx} for device D1. a, Measurements in zero field and **b,** for $\phi/\phi_0 = 1/2$. These data were used to calculate the mobilities and mean free paths in Fig. 1 of the main text.

Supplementary Note 4

BZ fermions at higher order fractions. In Fig. 1 of the main text, we presented μ and l for Dirac fermions and for BZ fermions at $\phi/\phi_0 = 1/2$. For completeness, Supplementary Fig. 4 shows the same analysis for the case of ϕ/ϕ_0 = 1/3 and 1/4. One can see that mobilities of BZ fermions with the larger q still remain of the order of 10^6 cm^2/Vs and their mean free path approaches values comparable to the device width W, which suggests a notable contribution from edge scattering.

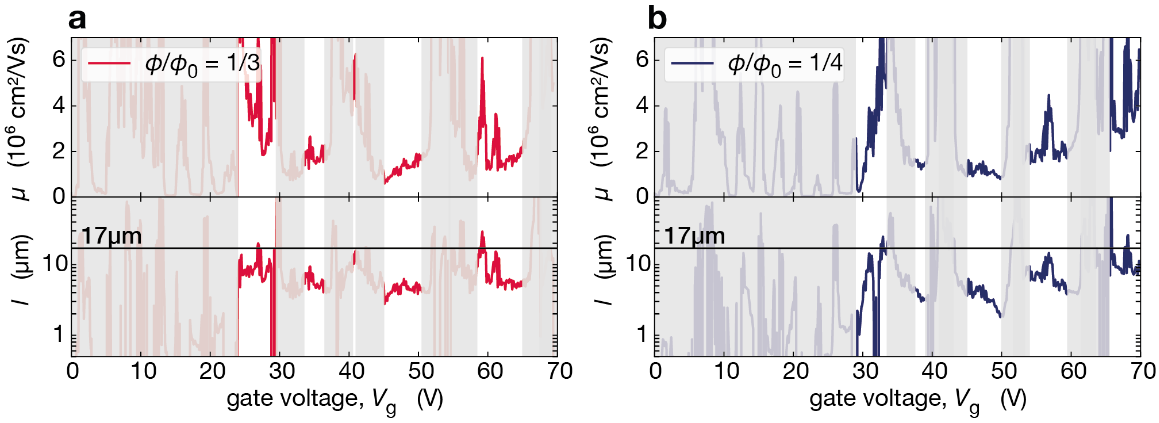

Supplementary Figure 4 | Ballistic transport of BZ fermions at unit fractions of the flux quantum. The data are for device D1 at 10 mK for $\phi/\phi_0 = 1/3$ (**a**) and $1/4$ (**b**). The same presentation as in Figs. 1b,d of the main text. We show the data for positive voltages because for mini-fans and vHS could accurately be identified only for electron doping.

Supplementary Note 5

Additional examples of ballistic transfer of BZ fermions. In the main text, we have emphasized that, at fields $B = B_{p/q}$, BZ fermions move through the superlattice as if the applied field were zero. The effective mass of BZ fermions depends on p/q because electronic spectra differ in different magnetic minibands. Away from the exact $B_{p/q}$ values, BZ fermions are expected[1] to experience an effective magnetic field $B_{eff} = B - B_{p/q}$ and, therefore, replicate magneto-transport effects known for charge carriers in conventional 2D electronic systems. This includes the negative bend resistance that is one of the most distinct, qualitative signatures of ballistic transport of charge carriers[5–7]. The effect can be understood as follows. With reference to Fig. 2a of the main text, let us for simplicity consider positive charge carriers (hole-doping regime). If holes injected from contact 3 can travel ballistically over a distance exceeding W (that is, can reach contact 1 without scattering), an extra positive charge would be accumulated near contact 1. As a result, the voltage difference $V_{21} = V_2 - V_1$ should be negative (see Fig. 2a of the main text). In contrast, if the transport is conventional (diffusive), holes from contact 3 travel along lines of the electric field and accumulate at contact 4. Accordingly, the sign of V_{21} should be conventional (that is, positive). The same consideration for V_{21} is valid for electrons. Therefore, the negative sign of R_b signifies ballistic transport over distances larger than W.

Negative R_b was reported in Fig. 2 of the main text for one of our devices (D2). Supplementary Fig. 5 provides further examples of ballistic transport of BZ fermions using two other superlattices (devices D3 and D4). Pronounced pockets of negative R_b are seen in Supplementary Fig. 5 at unit fractions of ϕ_0 with q from 2 to 5. Despite relatively small $W \approx 3$ µm, no evidence for ballistic transfer was observed for high-order BZ states ($p > 1$), in agreement with the results reported in the main text.

Note that occasionally we observed negative bend resistance away from $\phi/\phi_0 = 1/q$ (see, e.g., the vertical magenta stripe close to zero V_g in Supplementary Fig. 5a). Unlike the ballistic transfer resistance at unit flux fractions, negative signals away from the unit fractions were not reproducible in different contact configurations. Such 'extra' negative signals are not surprising in our experimental geometry and well known to appear in the quantum Hall effect regime using narrow (mesoscopic) devices[8].

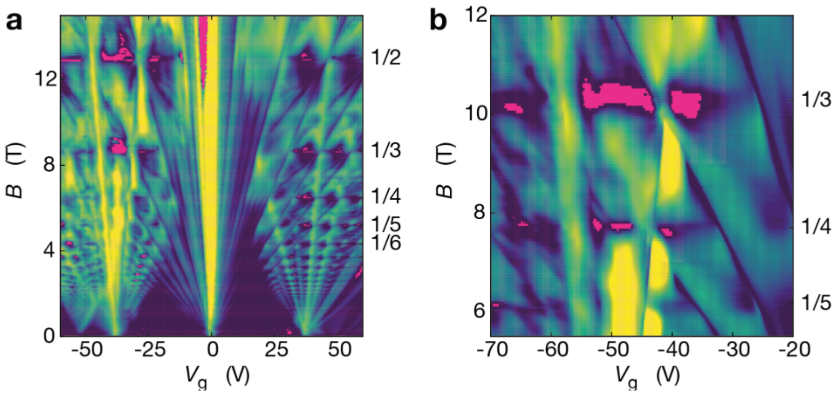

Supplementary Figure 5 | Ballistic transport of BZ fermions over micrometer distances. Fan diagrams obtained in the bend resistance geometry for devices D3 (**a**) and D4 (**b**) with $W = 3$ and 3.2 μm, respectively. $T = 2$ K. Pockets of negative R_b are highlighted in magenta. Indigo-to-yellow: Log scale truncated between 10 and 2,000 Ω to optimize the contrast.

Ballistic transport of BZ fermions was found to be rather sensitive to T, and the pockets of negative R_b universally disappeared above 30-50 K as shown in Supplementary Fig. 6. This is generally expected because the mean free path of BZ fermions should become shorter at higher T. However, the exact scattering mechanism could be nontrivial (see, e.g. Umklapp electron-electron scattering[9]) and requires further investigation.

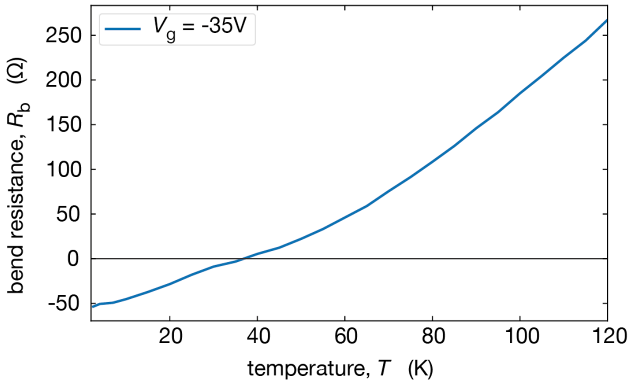

Supplementary Figure 6| Temperature dependence of BZ fermions' ballistic transport. An example of the bend resistance measured at $\phi/\phi_0 = 1/2$ using device D5 with W = 2 μm.

Supplementary Note 6

Supporting measurements in the longitudinal geometry. To crosscheck our conclusions about ballistic transport of BZ fermions, we compare the negative bend resistance measurements shown in Figs. 2c,d of the main text with those made in the conventional longitudinal geometry for the same device D2 (Supplementary Fig. 7a). The longitudinal resistance R_{xx} for BZ fermions was found positive in all the regions of the map where the negative bend resistance was reported, which corroborates the conclusion in the main text about ballistic transfer of BZ fermions across the device.

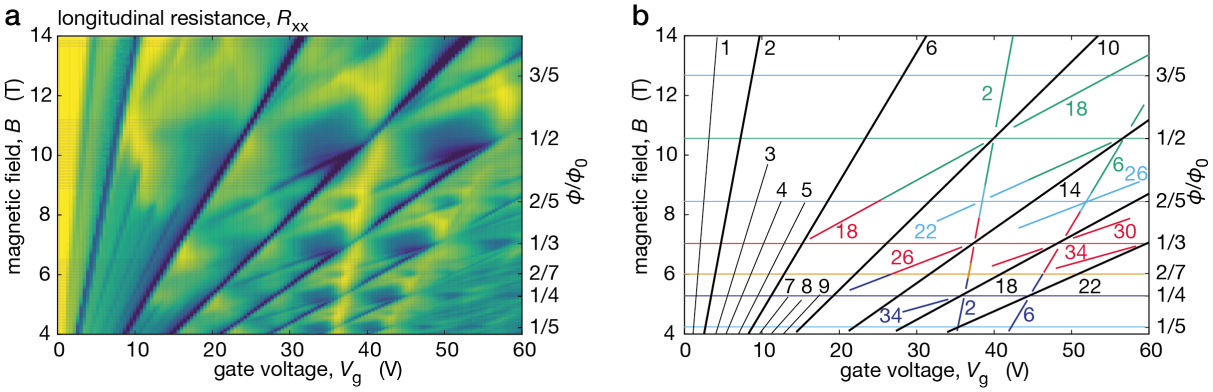

Supplementary Figure 7 | Longitudinal resistance for ballistic BZ fermions. a, R_{xx} as a function of gate voltage and magnetic field measured at 2 K for device D2. Color scale is the same as in Fig. 2c of the main text. **b**, Minima found in the longitudinal conductivity are shown schematically. The color-coding is the same as for device D1 in Fig. 3b of the main text. The thin black lines mark LLs with the lifted spin and valley degeneracy for Dirac fermions of the main spectrum.

Let us note here that, according to the group theory of irreducible representations for the group of translations in a magnetic field, an electronic spectrum for each realization of BZ fermions should have an additional q-fold degeneracy. This is prescribed by the fact that a group corresponding to any p/q fraction is non-Abelian (due to Aharonov-Bohm phases acquired upon translations in non-colinear directions) but contains an Abelian subgroup of translations corresponding to a magnetic superlattice with a q times larger supercell. The additional q-fold degeneracy takes the form of q mini-valleys in the magnetic mini Brillouin zone with an area q times smaller than the moiré superlattice Brillouin zone at $B = 0$. This degeneracy is additional to the 4-fold spin and valley degeneracy of graphene's original spectrum.

With this consideration in mind, the measurements in Supplementary Fig. 7 also support our other conclusion that the full degeneracy of BZ fermions is $4q$. Indeed, the q-fold degeneracy reported in Fig. 3 of the main text corresponds to the case where both spin and valley degeneracies of both Dirac and BZ fermions were lifted. Supplementary Fig. 7 shows LL fans at 2 K, the temperature much higher than 10 mK for the measurements in Fig. 3. Dirac fermions of the main spectrum exhibit the lifted spin and valley degeneracies by the relatively strong B (thin black lines in Supplementary Fig. 7b). At lower fields $B < 3$T, these interaction-induced gaps become progressively smeared. As for BZ fermions, their mini-fans visible in Supplementary Fig. 7b reach only the effective field $|B_{eff}| < 2$T, which does not allow the lifting of spin and valley degeneracies at this temperature. Accordingly, only the main sequence of LLs for BZ fermions could be observed at 2 K, and it corresponds to the $4q$-fold degeneracy, as expected and explained in the previous paragraph.

Supplementary Note 7

Lifting mini-valley degeneracy. In the main text we reported additional quantum Hall effect minima that cannot be explained within the single-particle Hofstadter-Wannier (dashed lines in Fig 3b of the main text). Those minima in σ_{xx} were attributed to BZ states with lifted mini-valley degeneracy. As an additional proof for the observed degeneracy lifting, Supplementary Fig. 8 shows measurements of Hall conductivity σ_{xy} for the relevant range of B and V_g where the dashed lines occur in Fig. 3b. One can see well developed plateaus with the quantized values that are fully consistent with the filling factors reported in the main text and marked in Fig. 3b. This observation strongly supports our conclusions about lifting of all the degeneracies of BZ fermions at low T.

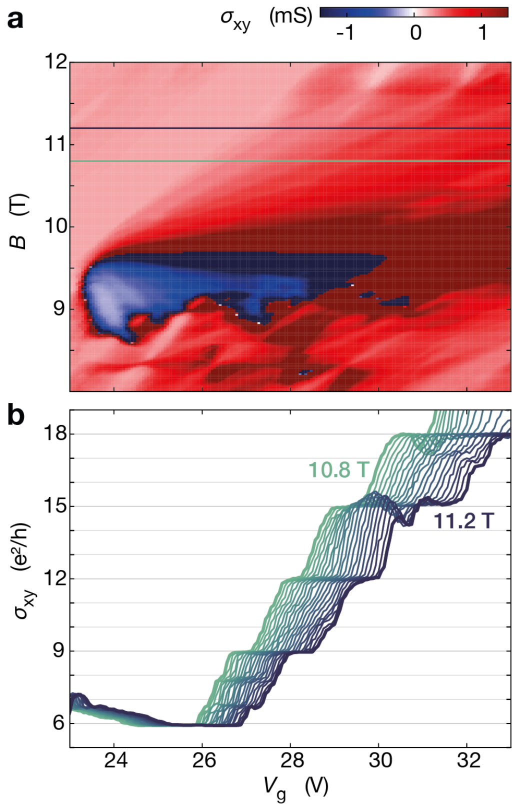

Supplementary Figure 8 | Quantized Hall conductance for BZ fermions. a, σ_{xy} around ϕ/ϕ_0= 1/3. **b**, Hall conductivity as a function of gate voltage at a number of constant *B* within the field interval around 11 T (color-coded). The interval is marked by the horizontal lines in (**a**).

The described lifting of mini-valley degeneracy involves very small energy gaps as witnessed by rapid disappearance of the corresponding features with increasing *T*. Indeed, the quantized Hall plateaus seen in the above figure and the conductance minima marked by the dashed lines in Fig. 3b of the main text could not be resolved at 2 K. The features also disappeared rapidly with increasing the excitation current. For example, Supplementary Fig. 9 shows a Landau mini-fan around ϕ/ϕ_0= 1/3 for currents of 10 and 100 nA. In the former case (Supplementary Fig. 9a), there are clear minima associated with to the lifted mini-valley degeneracy. The higher current (100 nA) resulted in complete smearing of these mini-gaps (Supplementary Fig. 9b), presumably because of an increase in the electronic temperature.

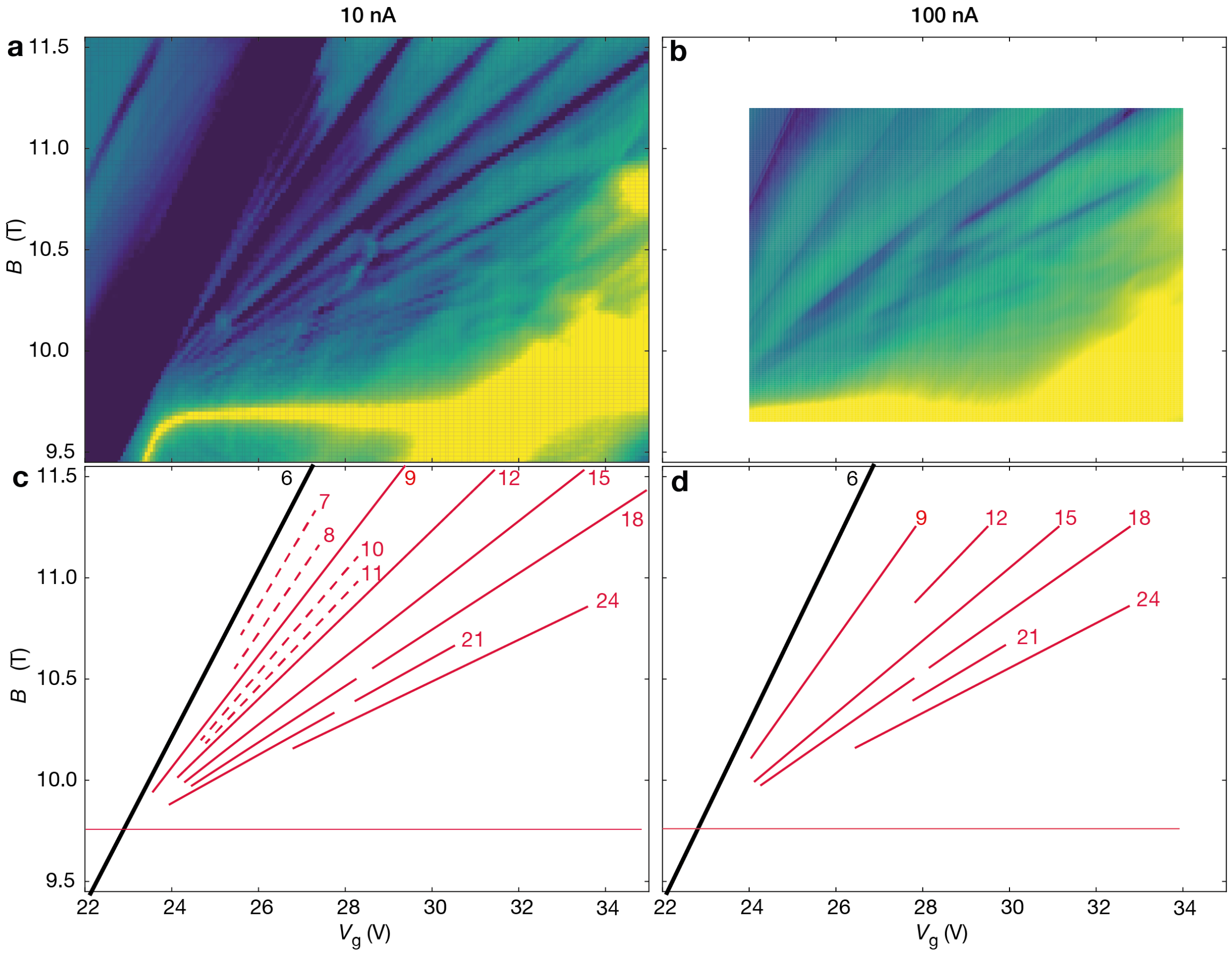

Supplementary Figure 9 | Landau mini-fans for different excitation currents. a and **b,** $\sigma_{xx}(B,V_g)$ at 10 mK for 10 and 100 nA, respectively. Indigo-to-yellow log scale: 310 nS to 780 µS. **c** and **d**, Minima found in (**a**) and (**b**) are shown schematically. The color-coded numbers are the filling factors for the nearby LLs. Thick black lines: Main sequence of LLs for graphene's Dirac spectrum.

Finally, let us draw attention to the rather unusual re-entrant behavior seen for the mini-fan around V_g = 28 V in Fig. 3 and Supplementary Fig. 9. The BZ-fermion gaps for ν = 18 and 21 seem to close within a certain interval B and V_g. We attribute this closure to competition between these BZ states and the ν = 7 state from the main Dirac sequence. The likely mechanism of the suppression of exchange gaps is discussed in ref. [10].

Supplementary References

1. Krishna Kumar, R. *et al.* High-temperature quantum oscillations caused by recurring Bloch states in graphene superlattices. *Science* **357**, 181–184 (2017).
2. Lee, M. *et al.* Ballistic miniband conduction in a graphene superlattice. *Science* **353**, 1526–1529 (2016).
3. Berdyugin, A. I. *et al.* Minibands in twisted bilayer graphene probed by magnetic focusing. *Sci. Adv.* **6**, eaay7838 (2020).
4. Kraft, R. *et al.* Anomalous Cyclotron Motion in Graphene Superlattice Cavities. (2020). preprint at arxiv:2006.14995
5. Beenakker, C. W. J. & van Houten, H. Quantum Transport in Semiconductor Nanostructures. in *Solid State Physics - Advances in Research and Applications* **44**, 1–228 (1991).
6. Beenakker, C. W. J. & van Houten, H. Billiard model of a ballistic multiprobe conductor. *Phys. Rev. Lett.* **63**, 1857–1860 (1989).
7. Gilbertson, A. M. *et al.* Ballistic transport and boundary scattering in InSb/In1-xAlx Sb mesoscopic devices. *Phys. Rev. B* **83**, 075304 (2011).

8. Büttiker, M. Negative resistance fluctuations at resistance minima in narrow quantum hall conductors. *Phys. Rev. B* **38**, 12724–12727 (1988).
9. Wallbank, J. R. *et al.* Excess resistivity in graphene superlattices caused by umklapp electron–electron scattering. *Nat. Phys.* **15**, 32–36 (2019).
10. Yu, G. L. *et al.* Hierarchy of Hofstadter states and replica quantum Hall ferromagnetism in graphene superlattices. *Nat. Phys.* **10**, 525–529 (2014).
